# Effects of intra-articular injection of platelet-rich plasma on the inflammatory process and histopathological characteristics of cartilage and synovium in animals with osteoarthritis: a systematic review with meta-analysis

**DOI:** 10.1186/s42358-024-00364-0

**Published:** 2024-03-29

**Authors:** Homero Garcia-Motta, Cristiano Carvalho, Evelyn Maria Guilherme, Marcos Paulo Braz de Oliveira, Karina Nogueira Zambone Pinto Rossi

**Affiliations:** 1 Morphology and Pathology Department, Federal University of São Carlos, São Carlos, Brazil; 2 Department of Biosciences, Federal University of São Paulo, Campus Baixada Santista, Rua Silva Jardim, 136, Santos, SP 11015-020 Brazil; 3 Physical Therapy Department, Federal University of São Carlos, São Carlos, Brazil

**Keywords:** Cartilage, Platelet-rich plasma, Synovitis, Interleukins, Tumor necrosis factor alpha

## Abstract

**Background:**

Osteoarthritis (OA) affects the entire joint, causing structural changes in articular cartilage, subchondral bone, ligaments, capsule, synovial membrane, and periarticular muscles that afflicts millions of people globally, leading to persistent pain and diminished quality of life. The intra-articular use of platelet-rich plasma (PRP) is gaining recognition as a secure therapeutic approach due to its potential regenerative capabilities. However, there is controversial clinical data regarding efficacy of PRP for OA treatment. In this context, gathering scientific evidence on the effects of PRP in treating OA in animal models could provide valuable insights into understanding its impact on aspects like cartilage health, synovial tissue integrity, and the inflammatory process in affected joints. Thus, the objective of this study was to assess the effects of PRP injections on inflammation and histopathological aspects of cartilage and synovium in animal models of OA through a comprehensive systematic review with meta-analysis.

**Methods:**

A electronic search was conducted on Medline, Embase, Web of Science, The Cochrane Library, LILACS, and SciELO databases for relevant articles published until June 2022. A random-effects meta-analysis was employed to synthesize evidence on the histological characteristics of cartilage and synovium, as well as the inflammatory process. The GRADE approach was utilized to categorize the quality of evidence, and methodological quality was assessed using SYRCLE’s RoB tool.

**Results:**

Twenty-one studies were included in the review, with twelve of them incorporated into the meta-analysis. PRP treatment demonstrated superior outcomes compared to the control group in terms of cartilage histology (very low quality; *p* = 0.0002), synovium histology (very low quality; *p* < 0.0001), and reductions in proinflammatory markers, including IL-1 (low quality; *p* = 0.002), IL-6 (very low quality; *p* < 0.00001), and TNF-α (very low; *p* < 0.00001). However, PRP treatment did not yield a significant impact on PDGF-A levels (very low quality; *p* = 0.81).

**Conclusion:**

PRP appears capable of reducing proinflammatory markers (IL-1, IL-6, TNF-α) and mitigating cartilage and synovium damage in animals with OA. However, the levels of evidence of these findings are low to very low. Therefore, more rigorous studies with larger samples are needed to improve the quality of evidence.

**PROSPERO registration:**

CRD42022250314

## Introduction

Osteoarthritis (OA) stands as one of the most widespread joint diseases [1], impacting an estimated 302 million individuals globally, including over 30 million in the United States alone [1, 2]. The well-being, both physical and mental, of those affected is significantly compromised due to pain, inflammation, and a decline in functionality [3, 4]. Beyond affecting public health systems, OA incurs socioeconomic costs through reduced work productivity and premature retirement [3, 5–7].

Treatment options for OA encompass surgical, non-pharmacological, and pharmacological approaches [8]. While surgical interventions are typically reserved for advanced stages when conservative treatments have proven ineffective [9], both non-pharmacological and pharmacological strategies are employed across mild and severe cases to alleviate pain, reduce joint stiffness, and preserve functionality [10]. Non-pharmacological methods include physical exercise, lifestyle adjustments, and self-management programs, which are strongly advocated for individuals with OA affecting the hand, hip, knee, or polyarthritis [2, 11–15]. Conversely, pharmacological approaches involve medications administered orally or via joint injection, with varying levels of recommendation [2, 15].

Intra-articular applications with platelet-rich plasma (PRP) have received attention in the treatment of OA [16, 17]. PRP is a blood product with a high platelet concentration obtained through the centrifugation of autologous venous blood [18, 19]. This enriched substance has a large diversity of growth factors (GFs) and other bioactive mediators [20, 21]. The regenerative potential of these substances is based on the functions of metabolic regulation, cell proliferation and extracellular matrix synthesis [22, 23]. With regard to cartilaginous tissue, a significant increase was found in the synthesis of extracellular matrix in chondrocytes treated with GFs [24]. Moreover, PRP proved to be efficient at reducing inflammatory markers and apoptosis in vivo [25, 26].

In spite of reported benefits, the Osteoarthritis Research Society International (OARSI) and the American College of Rheumatology (ACR) strongly advises against the intra-articular injection of PRP in individuals with OA. This caution is driven by the presence of low-quality evidence supporting its efficacy and the lack of standardization in its manufacturing process, encompassing variables such as the duration and speed of centrifugation, the use of anticoagulants and activators, and the concentration of platelets [2, 15, 27]. In this context, the compilation of scientific evidence regarding the effects of PRP in the treatment of OA in animal models could offer valuable insights into comprehending the impact of this therapeutic approach on various aspects such as cartilage health, synovial tissue integrity, and the inflammatory cascade within the affected joints. Therefore, the aim of systematic review with meta-analysis to investigate the effect of the intra-articular injection of PRP on the inflammatory process and histopathological characteristics of cartilage and synovium in animal models with OA.

## Methods

### Protocol and registration

The present review was conducted following the Preferred Reporting Items for Systematic Reviews and Meta-Analyses (PRISMA) [28] and the recommendations of the Cochrane Collaboration [29]. The quality of the evidence was assessed using the Grading of Recommendations, Assessment, Development and Evaluations (GRADE approach) [30].

The following question was used to guide this study: “How does the intra-articular injection of PRP affect the inflammatory process and histopathological characteristics of cartilage and synovium in animals with induced lesions aimed at developing OA?” To ensure a comprehensive analysis as well as the transparency of the methods and results, the protocol was registered in the International Prospective Register of Systematic Reviews (PROSPERO) (registration code: #CRD42022250314). As this was a systematic review of preclinical trials, there was no need for approval from an ethics committee.

### Eligibility criteria

#### Types of studies

Preclinical trials that evaluated the inflammatory process and histopathological characteristics following the intra-articular injection of PRP in animals with OA were included. Papers published in English, Portuguese and Spanish were considered.

#### Types of participants

Studies with any animal model that developed OA through surgical or pharmacological interference were included.

#### Types of comparators

Studies with comparison groups (control) treated either with a placebo or not submitted to any treatment were included.

#### Types of treatments

Studies that employed the intra-articular injection of PRP as the treatment were included. No restrictions were imposed regarding the dose, concentration, or production method of PRP.

#### Outcome measures

Studies reporting results related to changes (improvement, worsening or no change) in the inflammatory process (inflammatory markers) and/or histopathology (proliferation rate of chondrocytes and synoviocytes, synthesis of glycosaminoglycan (GAG), thickness of the cartilage and/or synovium) were included.

#### Exclusion criteria

Clinical trials, case studies, animal models with multiple diseases, in vitro or ex-vivo experiments and studies with control groups other than a placebo group or group without treatment were excluded.

### Development and data synthesis

#### Databases and search strategies

An electronic search was performed of the Medline, Embase, Web of Science, The Cochrane Library, LILACS and SciELO databases for relevant articles published up to June 2022. The search terms were selected considering the controlled vocabulary of the Medical Subject Headings (MeSH) database and uncontrolled vocabulary. The search strategy involved terms related to the topic of interest. Thus, the following combination of search terms was employed (“Platelet-Rich Plasma” OR “Platelet Gel” OR “Autologous Platelet Concentrate” OR “Autologous Conditioned Plasma” OR ACP) AND (Osteoarthritis) AND (Animals OR “Models, Animal” OR “Animal Experimentation”) AND (Inflammation OR “Intercellular Signaling Peptides and Proteins” OR Cartilage OR “Synovial Membrane”). A manual search was conducted by screening the reference lists of the studies included to identify potentially relevant studies not retrieved during the electronic search.

#### Selection of studies

Two independent reviewers (C.C. and H.G.M.) selected titles and abstracts of publications encountered during the electronic search based on the inclusion criteria. Potentially relevant studies were preselected for full-text analysis. The entire selection process was conducted by consensus. When a consensus was not reached, a third reviewer (K.N.Z.P.R.) was consulted to make the final decision. The StArt (State of the Art through Systematic Review) reference management software was used during the selection of the studies [31]. The StArt software automatically detected duplicates.

#### Data extraction

After the selection of the studies, the reviewers (C.C. e H.G.M.) worked independently. A standard form adapted from the model proposed by the Cochrane Collaboration was used to extract data on the study design, characteristics of the animals, treatment and comparison groups and outcomes [29].

#### Appraisal of methodological quality

The methodological quality was assessed using the SYRCLE’s risk of bias (RoB) tool for animal studies [32], analyzing risks related to selection, performance, detection, attrition and other biases. Two reviewers (E.M.G. and H.G.M.) scored the items independently, with disagreements resolved by a third reviewer (C.C.).

#### Data synthesis and analysis

The quality of the scientific evidence was analyzed using the GRADE approach, which has the following domains: limitations (risk of bias), inconsistency, indirectness, imprecision and publication bias [30]. The item 1 (limitations) was classified as “serious” when less than 75% of the studies included in comparison group fulfilled less than three items of the SYRCLE’s RoB tool. Meta-analyses were conducted using the RevMan 5 software [Review Manager 5.4 (RevMan)] [33]. Effect sizes were calculated using standardized mean differences (SMD) and with 95% confidence intervals (CI). The random effects models were used to calculate the pooled mean effect size. The effect size was classified as small (< 0.20), moderate (0.21 to 0.79) or large (> 0.80). The I^2^ statistic was used to assess heterogeneity among studies by comparison groups of meta-analyses, with values of ≥ 25, 50 and 75% interpreted as representing low, moderate and high heterogeneity, respectively [34].

## Results

### Description of studies

**Fig. 1:**
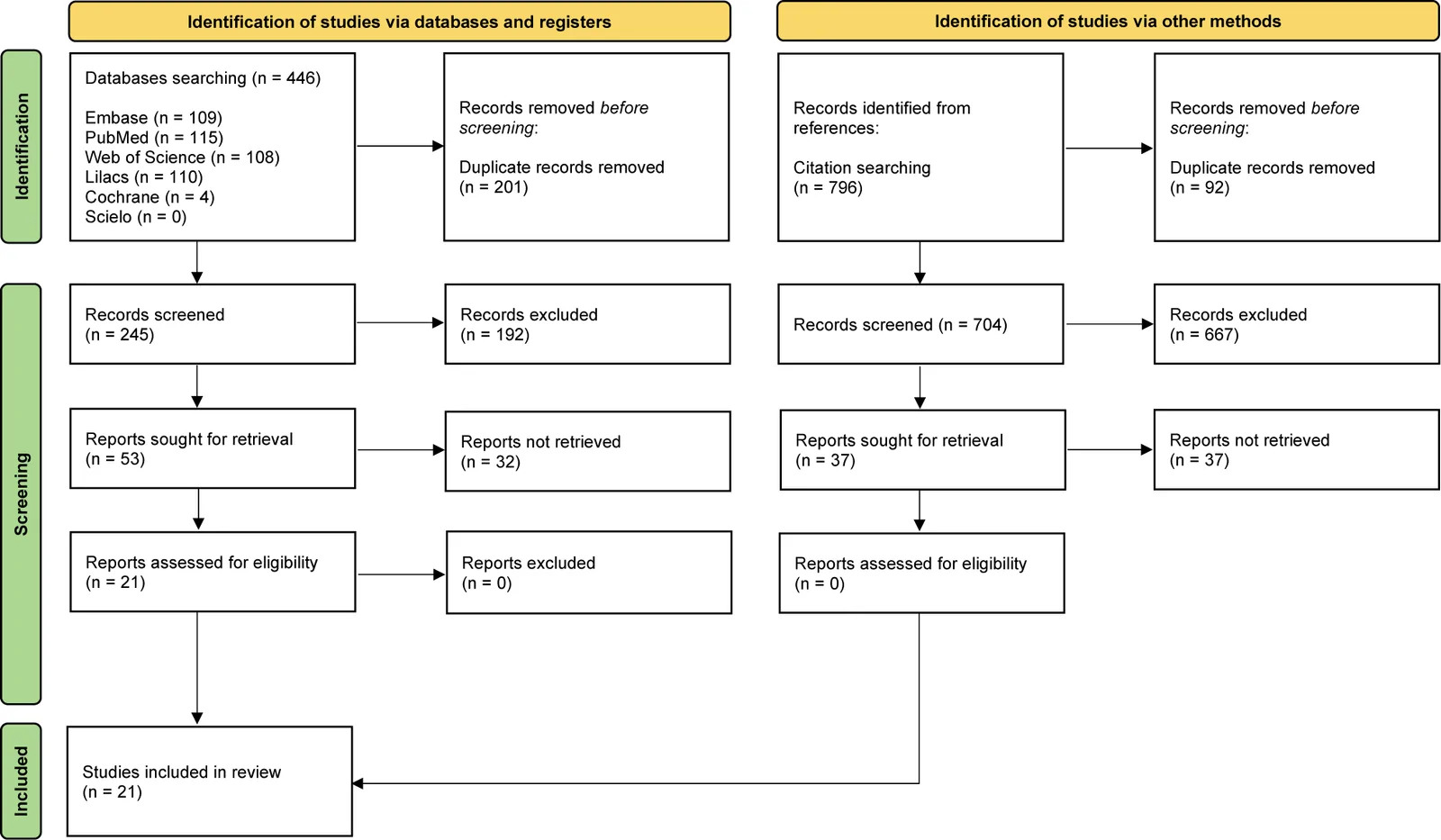
Flow diagram of the selection process

The electronic search of the databases led to the retrieval of 446 studies. After the selection process performed by consensus, 21 studies [16, 25, 26, 35–52] were included in the present review, involving a total of 456 animal joints (243 in the PRP-treated group and 213 in the control group). Twelve studies were included on meta-analyses and the assessment of the quality of the evidence by GRADE approach [16, 25, 36, 37, 39, 41, 44–46, 48, 50, 52]. The details of the selection process and main reasons for exclusions of studies are presented in Fig. 1.

### Characteristics of studies

The main characteristics of the 21 studies included are displayed in Table 1. The number of animals in the comparison groups ranged from 5 [43, 48, 50] and 16 [40, 49] and the average number of animals was 10. Different animal models were used in the studies included. Ten studies used a rodent model: three used Sprague-Dawley rats [16, 25, 43], four used albino rats [26, 46, 50, 51], two used Dunkin-Hartley guinea pigs [37, 48] and one used FVB/N mice [35]. Among those with a non-rodent model, seven studies used New Zealand rabbits [36, 38, 39, 42, 43, 49, 52], three used dogs [40, 45, 47] and one used Boer goats [41]. The vast majority of studies included (90%) used the knee as the target of the PRP treatment [16, 25, 35–37, 39–48, 50–52]. Two studies (9%) used the temporomandibular joint [38, 49] and one study (4,7%) used the ankle joint [26].

**Table 1:** Summary of descriptive characteristics of the included studies

Study	Population		Comparison groups	Intervention		Outcomes				
Authors (year)	Animal model strain, sex, weight, age	OA induction	(number)	Dose of PRP	Treatment protocol	Variable	Tool	Statistical results	*p*-value	Effect size (95% confidence interval)
Ahmad et al. (2020)	Rat Sprague Dawley N/A 400–550 g 4–5 mo.	Anterior cruciate ligament transection and Medial Meniscectomy	CG (10) PRP Group (10)	1 mL of PRP	Single injection	Inflammatory markers (TNF-α levels) Inflammatory markers (IL-6 levels) Molecular markers (COL2 levels) Molecular markers (proteoglycans) Cartilage histology, Mankin Score	ELISA ELISA N/A Semi-quantitative analysis N/A	+ + + + =	*p* = 0.039 *p* = 0.003 *p* = 0.12 *p* = 0.035 *p >* 0.05	2.45 (4.87 to 10.93) 5.09 (26.42 to 38.38) N/A 2.55 (−9.17 to −4.23) 0.95 (0.01 to 2.19)
Almasry et al. (2014)	Albino rat Wistar 25 *♂* 250–350 g Adult	Medial Meniscectomy	CG (12) PRP Group (13)	0.085 mL of PRP	3x/wk. for 2 wk.	Molecular markers (PDGF-A levels) Molecular markers (VEGF-A levels) Molecular markers (PDGF-A localization) Molecular markers (VEGF-A localization) Cartilage histology (Matrix loss) Cartilage histology (Degeneration) Cartilage histology (Zonal depth ratio of lesions) Cartilage histology (Total degeneration width) Cartilage histology (Significant degeneration width) Cartilage histology (Osteophyte) Cartilage histology (Medial capsule width) Cartilage histology (Subchondral bone damage) Synovium histology (Synovial reaction)	ELISA ELISA Quantitative analysis Quantitative analysis Modified OARSI Score Modified OARSI Score Modified OARSI Score Modified OARSI Score Modified OARSI Score Modified OARSI Score Modified OARSI Score Modified OARSI Score Modified OARSI Score	+ + + + + + + + + + + + +	*p* < 0.001 *p* < 0.001 *p* = 0. 007 *p* = 0. 002 *p* = 0.001 *p* < 0.001 *p* < 0.001 *p* < 0.001 *p* = 0.001 *p* = 0.041 *p* < 0.001 *p* < 0.001 *p* < 0.003	6.43 (22.04 to 28.56) 14.97 (43.64 to 48.76) 4.89 (7.92 to 11.16) 5.67 (6.46 to 8.72) −7.44 (−618.41 to −464.54) N/A −9.00 (−0.98 to −0.82) −8.75 (−673.87 to −555.13) −7.23 (−460.29 to −375.71) N/A −7.51 (−105.47 to −84.53) N/A N/A
Arican et al. (2018)	Dog Mixed breed N/A 25–50 kg 8–10 yr.	Naturally occurring	CG (6) PRP Group (14)	4–5 mL of PRP	Single injection	Molecular markers (MMP-2) Molecular markers (MMP-9)	ELISA ELISA	= +	*p >* 0.05 *p* < 0.05	N/A N/A
Asjid et al. (2018)	Rat Sprague Dawley N/A 200–300 g 3–4 mo.	MIA injection	CG (8) PRP Group (8)	0.5 mL of PRP	Single injection	Cartilage histology	Mankin Score	+	*p* = 0.003	−1.78 (−3.20 to −0.80)
Chouhan et al. (2019)	Guinea pig Dunkin-Hartley N/A 600–800 g 5 mo.	Naturally occurring	CG A (5) CG B (5) S. PRP Group A (5) M. PRP Group A (6) S. PRP Group B (5) M. PRP Group B (6)	Unclear	Single injection (S) 1x/wk. for 3 wk. (M)	CG A Vs. S. PRP Group A Synovium histology Cartilage histology CG A Vs. M. PRP Group A Synovium histology Cartilage histology CG B Vs. S. PRP Group B Synovium histology Cartilage histology CG B Vs. M. PRP Group B Synovium histology Cartilage histology	Pelletier Score Mankin Score Pelletier Score Mankin Score Pelletier Score Mankin Score Pelletier Score Mankin Score	+ = + = + = + =	*p* = 0.011 *p* = 0.442 *p* = 0.002 *p* = 0.005 *p* = 0.562 *p* ≥ 0.999 *p* = 0.000 *p* = 0.146	−0.19 (−3.46 to 2.66) 0.08 (−3.66 to 4.06) -0.46 (−4.8 to 2.40) -0.90 (−6.14 to 1.28) 1.54 (0.11 to 4.29) 1.42 (−0.13 to 8.93) -0.01 (−3.15 to 3.09) 0.43 (−1.26 to 2.40)
Coskun et al. (2018)	Rabbit New Zealand 32 *♂* 3–3,5 kg Adult	MIA injection	S. CG (8) M. CG (8) S. PRP Group (8) M. PRP Group (8)	0.8–1 mL of PRP	Single injection (S) 1x/wk. for 3 wk. (M)	S. CG Vs. S. PRP Group Cartilage histology (Cartilage structure) Cartilage histology (Osteochondral junction) Cartilage histology (Chondrocyte appearance) Cartilage histology (Subchondral bone structure) M. CG Vs. M. PRP Group Cartilage histology (Cartilage structure) Cartilage histology (Osteochondral junction) Cartilage histology (Chondrocyte appearance) Cartilage histology (Subchondral bone structure)	Qualitative Score Qualitative Score Qualitative Score Qualitative Score Qualitative Score Qualitative Score Qualitative Score Qualitative Score	= = = = = = = =	*p >* 0.05 *p >* 0.05 *p >* 0.05 *p >* 0.05 *p >* 0.05 *p >* 0.05 *p >* 0.05 *p >* 0.05	N/A N/A N/A N/A N/A N/A N/A N/A
Gamal et al. (2019)	Albino rat Wistar 15 *♂* 200–250 g 4–5 mo.	Cartilage defect surgical procedure	CG A* (5) CG B* (5) PRP Group (5)	0.2 mL of PRP	3x/wk. for 2 wk.	Molecular markers (PDGF-A levels) Cartilage histology (thickness)	Immunohistochemical analysis Qualitative analysis	+ +	*p* < 0.05 *p* < 0.05	9.02 (−12.88 to −3.39) 33.81 (333.57 to 363.63)
Guner & Buyukbebeci (2013)	Albino rat Wistar 20 *♂* 250–300 g Adult	Formalin injection	CG (10) PRP Group (10)	0.05 mL of PRP	1x/wk. for 3 wk.	Cartilage histology	Degeneration Score	=	*p >* 0.05	N/A
Hermeto et al. (2016)	Rabbit New Zealand 12 *♂* 3.5–4,5 kg Adult	Collagenase injection	CG (6) PRP Group (6)	Unclear	Unclear	Cartilage histology	Modified Mankin Score	=	*p >* 0.05	−3.00 (−6.67 to −2.67)
Jayaram et al. (2020)	Mice FVB/N 33 *♂* N/A 3 mo.	Destabilization of the Medial Meniscus	CG (10) LP-PRP Group (11) LR-PRP Group (12)	0.005 mL of PRP	3 injections	CG Vs. LP-PRP Group Cartilage histology Synovium histology CG Vs. LR-PRP Group Cartilage histology Synovium histology	OARSI Score Synovitis Score OARSI Score Synovitis Score	= = = =	*p* = 0.3157 *p* = 0.9999 *p* = 0.9999 *p* = 0.9999	N/A (−23.96 to 3.66) N/A N/A (18.50 to 9.120) N/A
Ji et al. (2015)	Rabbit New Zealand N/A ♀ N/A *♂* 1.75–2.75 kg 58 mo.	Modified Hulth Protocol	CG (10) PRP Group (11)	0.5 mL of PRP	1x/wk. for 5 wk.	Inflammatory markers (IL-1 levels) Inflammatory markers (IL-6 levels) Inflammatory markers (TNF-α levels)	ELISA ELISA ELISA	+ + +	*p* < 0.05 *p* < 0.05 *p* < 0.05	0.64 (−1.50 to 0.26) 2.43 (−3.50 to −1.17) 3.24 (−4.47 to −1.76)
Kanwat et al. (2017)	Guinea pig Dunkin-Hartley 24 *♂* 600–700 g N/A	Naturally occurring	CG A (6) CG B (6) PRP Group A (6) PRP Group B (6)	Unclear	1x/wk. for 3 wk.	CG A Vs. PRP Group A Synovium histology Cartilage histology CG B Vs. PRP Group B Synovium histology Cartilage histology	Pelletier Score Modified Mankin Score Pelletier Score Modified Mankin Score	= + + +	*p* = 0.147 *p* = 0.002 *p* = 0.001 *p* = 0.0001	−1.33 (−6.23 to −0.11) −2.45 (−1.76 to 1.08) −2.50 (−7.57 to −2.43) −3.00 (−9.91 to −3.09)
Kutuk et al. (2014)	Rabbit New Zealand N/A 2.64–3 kg 12 mo.	Bilateral TMJ surgical procedure	CG (10) PRP Group (10)	1 mL of PRP	Unclear	Cartilage histology (Fibrocartilage regeneration) Cartilage histology (Regeneration hyaline cartilage)	Quantitative analysis Quantitative analysis	= =	*p* = 0.143 *p* = 0.579	N/A N/A
Lu et al. (2020)	Rabbit New Zealand 17 ♀ 13 *♂* 3.0–4.5 kg ± 11 wk.	Modified Hulth Protocol	CG (15) PRP Group (15)	0.5 mL of PRP	1x/wk. for 10 wk.	Inflammatory markers (IL-1 levels) Inflammatory markers (IL-6 levels) Inflammatory markers (TNF-α levels) Cartilage histology	ELISA ELISA ELISA Mankin Score	+ + + +	*p* < 0.01 *p* < 0.01 *p* < 0.01 *p* < 0.05	−2.60 (−23.75 to −13.13) −4.50 (−54.56 to −39.00) −5.93 (−74.08 to −57.48) N/A
Parlak & Arican (2020)	Dog Mixed breed 6 ♀ 30 *♂* 29–31 kg 4–6 yr.	Naturally occurring	CG (16) S. PRP Group (8) M. PRP Group (8)	Unclear	Single injection (S) 1x/wk. for 2 wk. (M)	Inflammatory markers (TNF-α levels) Inflammatory markers (IL-10 levels) Inflammatory markers (IL-6 levels) Inflammatory markers (IL-1β levels)	ELISA ELISA ELISA ELISA	= + + +	*p >* 0.05 *p* < 0.05 *p* < 0.05 *p* < 0.05	N/A N/A N/A N/A
Ragab et al. (2021)	Albino rat Wistar 20 *♂* 100–120 g 7–9 wk.	MIA injection	CG (10) PRP Group (10)	0.05 mL of PRP	1x/wk. for 3 wk.	Anti-inflammatory markers (IL-4 levels) Inflammatory markers (IL-17 levels) Inflammatory markers (TNF-α levels)	ELISA ELISA ELISA	+ + +	*p* < 0.05 *p* < 0.05 *p* < 0.05	N/A N/A N/A
Wang et al. (2018)	Goat Boer 12 *♂* 40–50 kg 12–18 mo.	Anterior cruciate ligament transection and Medial Meniscectomy	CG (6) PRP Group (6)	2 mL of PRP	3x every 4 wk.	Inflammatory markers (IL-1β levels) Inflammatory markers (IL-6 levels) Inflammatory markers (TNF-α levels) Molecular markers (PGE2 levels) Cartilage histology	ELISA ELISA ELISA ELISA Pelletier Score	+ = = + +	*p* < 0.05 *p >* 0.05 *p >* 0.05 *p* < 0.05 *p* < 0.05	N/A N/A N/A N/A N/A
Wu et al. (2016)	Rabbit New Zealand 32 ♀ 28 *♂* 2.5–3.0 kg 4–5 mo.	Modified Hulth Protocol	CG (15) PRP Group (15)	0.5 mL of PRP	1x/wk. for 6 wk.	Inflammatory markers (IL-1β levels in the joint fluid) Inflammatory markers (IL-1β levels in the serum) Inflammatory markers (IL-1β positive chondrocytes Inflammatory markers (Density of IL-1β staining) Cartilage histology Cartilage histology	ELISA ELISA Immunohistochemical analysis Immunohistochemical analysis Mankin Score Pelletier Score	+ + + + + +	*p* = 0.0079 *p* = 0.0086 *p* = 0.0068 *p* = 0.0073 *p* = 0.0081 *p* < 0.05	N/A N/A N/A N/A N/A N/A
Xin et al. (2020)	Rat Sprague Dawley N/A 200–300 g 3–4 mo.	Anterior cruciate ligament transection	CG (5) PRP Group (5)	0.05 mL of PRP	1x/wk. for 4 wk.	Inflammatory markers (IL-1β levels) Inflammatory markers (IL-18 levels) Inflammatory markers (TNF-α levels) Molecular markers (Col2 levels) Cartilage histology Cartilage histology	ELISA ELISA ELISA ELISA Mankin Score OARSI Score	+ + + + + +	*p* < 0.05 *p* < 0.05 *p* < 0.05 *p* < 0.05 *p* < 0.01 *p* < 0.01	N/A N/A N/A N/A N/A N/A
Yin et al. (2016)	Rabbit New Zealand 25 ♀ 25 *♂* 2.5–3.0 kg Adult	Anterior cruciate ligament transection	CG (10) PRP Group (10)	3 mL of PRP	1x/wk. for 3 wk.	Inflammatory markers (IL-1β levels) Molecular markers (PGE2 levels)	ELISA ELISA	+ +	*p* < 0.05 *p* < 0.05	N/A N/A
Yun et al. (2016)	Dog Beagle 6 ♀ 30 *♂* 29–31 kg 4–6 yr.	Cranial cruciate ligament transection	CG (6) PRP Group (6)	1 mL of PRP	1x/wk. for 4 wk.	Femoral cartilage (TNF-α levels) Femoral cartilage (IL-1β + levels) Tibial cartilage (TNF-α levels) Tibial cartilage (IL-1β + levels) Femoral cartilage (Surface damage) Femoral cartilage (Hypocellularity) Femoral cartilage (Clone) Femoral cartilage (Stain intensity) Tibial cartilage (Surface damage) Tibial cartilage (Hypocellularity) Tibial cartilage (Clone) Tibial cartilage (Stain intensity)	Immunohistochemical analysis Immunohistochemical analysis Immunohistochemical analysis Immunohistochemical analysis Mankin Score Mankin Score Mankin Score Mankin Score Mankin Score Mankin Score Mankin Score Mankin Score	+ + + + + + + + + + + +	*p* < 0.01 *p* < 0.01 *p* < 0.01 *p* < 0.01 *p* < 0.01 *p* < 0.01 *p >* 0.05 *p >* 0.05 *p* < 0.01 *p >* 0.05 *p >* 0.05 *p >* 0.05	−6.29 (−115.62 to 76.38) −7.14 (−152.65 to 106.03) −2.83 (−134.09 to −50.25) −4.21 (−170.39 to −90.61) −3.44 (−2.29 to −1.05) −1.79 (−1.44 to −0.24) −0.77 (−1.33 to −0.33) −1.82 (−1.14 to −0.20) −3.44 (−2.29 to −1.05) −2.22 (−2.12 to −0.56) −0.57 (−1.07 to −0.41) −0.68 (−0.95 to −0.29)

*Note* The effect size and confidence interval were calculated for each outcome considering the sample as well as the mean and standard deviation values of the PRP-treated and comparison groups

*Abbreviations*: *♀* female; *♂* male; *CG* control group; *CG A* control group analyzed at three months; CG A* control group analyzed at four weeks; *CG B* control group analyzed at six months; CG B* control group analyzed at six weeks; *COL2* collagen 2; *ELISA* enzyme-Linked immunosorbent assay; *IL-1* interleukin 1; *L-1β* interleukin 1β; *IL-6* interleukin 6; *IL-10* interleukin 10; *M. CG* multiple injections control group; *M.PRP Group A* multiple injections intervention group analyzed at three months; *M.PRP Group B* multiple injection intervention group analyzed at six months; *MMP-2* matrix metalloproteinase-2; *MMP-9* matrix metallopeptidase-9; *MMP-13* matrix metallopeptidase-13; *MIA* monosodium iodoacetate; *Mo.* months; *N/A* not available; *OA* osteoarthritis; *OARSI* osteoarthritis research society international; *PBS* phosphate buffered saline; *PDGF* platelet-derived growth factor; *PGE2* prostaglandin E2; *PRP* platelet-rich plasma; *PRP Group A* intervention group analyzed at three months; *PRP Group B* intervention group analyzed at six months; *S. CG* single injection control group; *S.PRP Group A* single injection intervention group analyzed at three months; *S.PRP Group B* single injection intervention group analyzed at six months; *TMJ* temporomandibular joint; *TNF-α* tumor necrosis factor alpha; *VEGF* vascular endothelial growth factor; *Wk.* week; *Yr.* years

For the induction of OA, surgical procedures were performed in 12 of the 21 studies included [25, 35, 36, 38, 39, 41–46, 50]. Chemical methods were employed in five studies [16, 26, 49, 51, 52]. Four studies used animal models that developed OA naturally and therefore did not require any induction method [37, 40, 47, 48]. The different surgical procedures involved a meniscus transection [35, 46], sectioning of ligaments around the knee joint [43–45], a combination of both methods [25, 41], a bilateral destabilization of the temporomandibular joint [38] or the modified Hulth Protocol [36, 39, 42]. The chemical method most adopted was the injection of monosodium iodoacetate (MIA), which was used in three studies [16, 26, 49], followed by the injection of collagenase [52]. A formalin solution was used in one study [51].

Although the PRP preparation method is limited to the double-centrifugation method or kits developed by specialized companies, no standardization in the manufacturing steps of the blood product was found with regards to centrifugation time or speed, preparation temperature, the administration of activators or anticoagulants or platelet count (Table 2). The range of the platelet concentration reported in 13 studies was three to eight times higher than the normal concentration in blood [16, 35, 37, 38, 40–45, 47, 48, 52]. Divergences were also found regarding the dose of PRP applied, even in studies using the same species of animal. For instance, the dose in the experiments conducted in two studies [42, 44] had a difference of 2.5 mL, although both experimental units shared the same characteristics. Likewise, the dose administration regime diverged considerably. The number of applications ranged from one [16, 25, 47–49] to ten [39] and the frequency ranged from a single time [26, 36, 37, 39, 40, 42–45, 48, 49, 51] to up to three times per week [41, 46, 50].

**Table 2:** Summary platelet-rich plasma preparation methods

Platelet-rich plasma preparation												
Study	Preparation protocol	1st centrifugation			2nd centrifugation			Cell count			Supplementation	
Authors		Time	Spin speed		Time	Spin speed		Platelets	**Leukocytes (**×10^6^)		Anticoagulant	Activation
Ahmad et al.	Double centrifuge method	12 min	300 g		8 min	4000 rpm		N/A	N/A		N/A	10% calcium gluconate
Almasry et al.	Double centrifuge method	20 min	160 g		15 min	400 g		N/A	N/A		0.5 mL of 0.1 M sodium citrate	0.05 mL of 0.2 M calcium chloride
Arican et al.	Double centrifuge method	10 min	96 g		20 min	380 g		1,420,000/μL	1.09 s/μL		Sodium citrate	N/A
Asjid et al.	Double centrifuge method	10 min	500 g		10 min	2200 g		3 times greater than baseline	N/A		0.05 mL of 0.1 M sodium citrate	50 μL of 10% calcium chloride
Chouhan et al.	Double centrifuge method	20 min	800 rpm		15 min	2200 rpm		≅ 3 times greater than baseline	Low (Unclear)		Acid citrate dextrose	N/A
Coskun et al.	Standard PRP kit	N/A	N/A		N/A	N/A		N/A	N/A		Sodium citrate	Bovine thrombin and calcium chloride
Gamal et al.	Double centrifuge method	8 min	1240 rpm		5 min	1240 rpm		N/A	N/A		Sodium citrate	N/A
Guner & Buyukbebeci	Double centrifuge method	10 min	5600 rpm		10 min	2400 rpm		N/A	N/A		EDTA	N/A
Hermeto et al.	Double centrifuge method	10 min	300 g		10 min	640 g		997.42 ± 48.01/ml	N/A		10% sodium citrate	0.05 mL 10% calcium gluconate
Jayaram et al.	Double centrifuge method	6 min	1500 rpm		15 min	3400 rpm		LP-PRP: 1.556 × 10^6^ ± 0.0216 × 10^6^ LR-PRP: 2.760 × 10^6^ ± 0.0898 × 10^6^	LP-PRP: 0.970 ± 0.0361 LR-PRP: 3.210 ± 0.6322		100 μL of citrate dextrose solution	N/A
Ji et al.	Unclear	15 min	2000 rpm		-	-		N/A	N/A		0.4 mL of citrate glucose	0.02 mL calcium chloride
Kanwat et al.	Double centrifuge method	20 min	800 rpm		15 min	2200 rpm		G1: 1,820,000/μL G2: 1,963,000/μL (≅ 3 times greater than the baseline)	N/A		Acid citrate dextrose	N/A
Kutuk et al.	Standard PRP kit	Unclear	Unclear		Unclear	Unclear		5.24 times greater than the baseline	N/A		2 mL of citrate dextrose	10% calcium chloride
Lu et al.	Double centrifuge method	10 min	1500 g		Unclear	Unclear		N/A	N/A		N/A	N/A
Parlak & Arican	Standard PRP kit	5 min	1700 g		Unclear	Unclear		4N/A8 times greater than the baseline	N/A		3 mL of acid citrate	N/A
Ragab et al.	Double centrifuge method	10 min	1000 rpm		10 min	2000 rpm		N/A	N/A		3.8% sodium citrate	50 μL 10% calcium chloride
Wang et al.	Standard PRP kit	10 min	350 g		15 min	450 g		1330.1 × 10^3^/mL	N/A		10 mL acid citrate dextrose	CaCl_2_
Wu et al.	Double centrifuge method	10 min	215 g		10 min	863 g		1958.33 ± 316.41 × 10^9^/L	N/A		1 mL of 10% sodium citrate	N/A
Xin et al.	Double centrifuge method	10 min	150 g		10 min	1500 g		1:0 × 10^9^–2:0 × 10^9^/mL	N/A		Acid citrate dextrose	N/A
Yin et al.	Double centrifuge method	10 min	250 g		10 min	1000 g		1947.50 ± 297.71 × 10^6^	56.92 ± 26.83		Acid citrate dextrose	N/A
Yun et al.	Double centrifuge method	10 min	1200 rpm		10 min	2500 rpm		> 1.0 × 10^6^/μL	N/A		7 mL of acid citrate dextrose	N/A

*Note*: *N/A* not available

The outcomes were evaluated using a variety of methods. Sixteen studies evaluated cartilage histology and/or synovium histology to investigate tissue regeneration [16, 25, 35, 37–39, 41–43, 45, 46, 48–52]. Thirteen studies investigated the inflammatory process by determining the expression of inflammatory markers or other molecules affected during joint inflammation, such as COL-2, MMP13, PDGF-A and VEGF [25, 26, 36, 39–47, 50]. The main classification systems used in the histological evaluations were the Modified Mankin Score [53] in nine studies [16, 25, 38, 41, 43, 45, 46, 48, 52], Pelletier [54] in four studies [37, 41, 42, 48] and OARSI [55] in three studies [35, 43, 46]. The most common immunodiagnostic method was ELISA test in eleven studies [25, 26, 36, 39–44, 46, 47], followed by immunohistochemical analysis in three studies [42, 45, 50].

### Appraisal of methodological quality

**Fig. 2:**
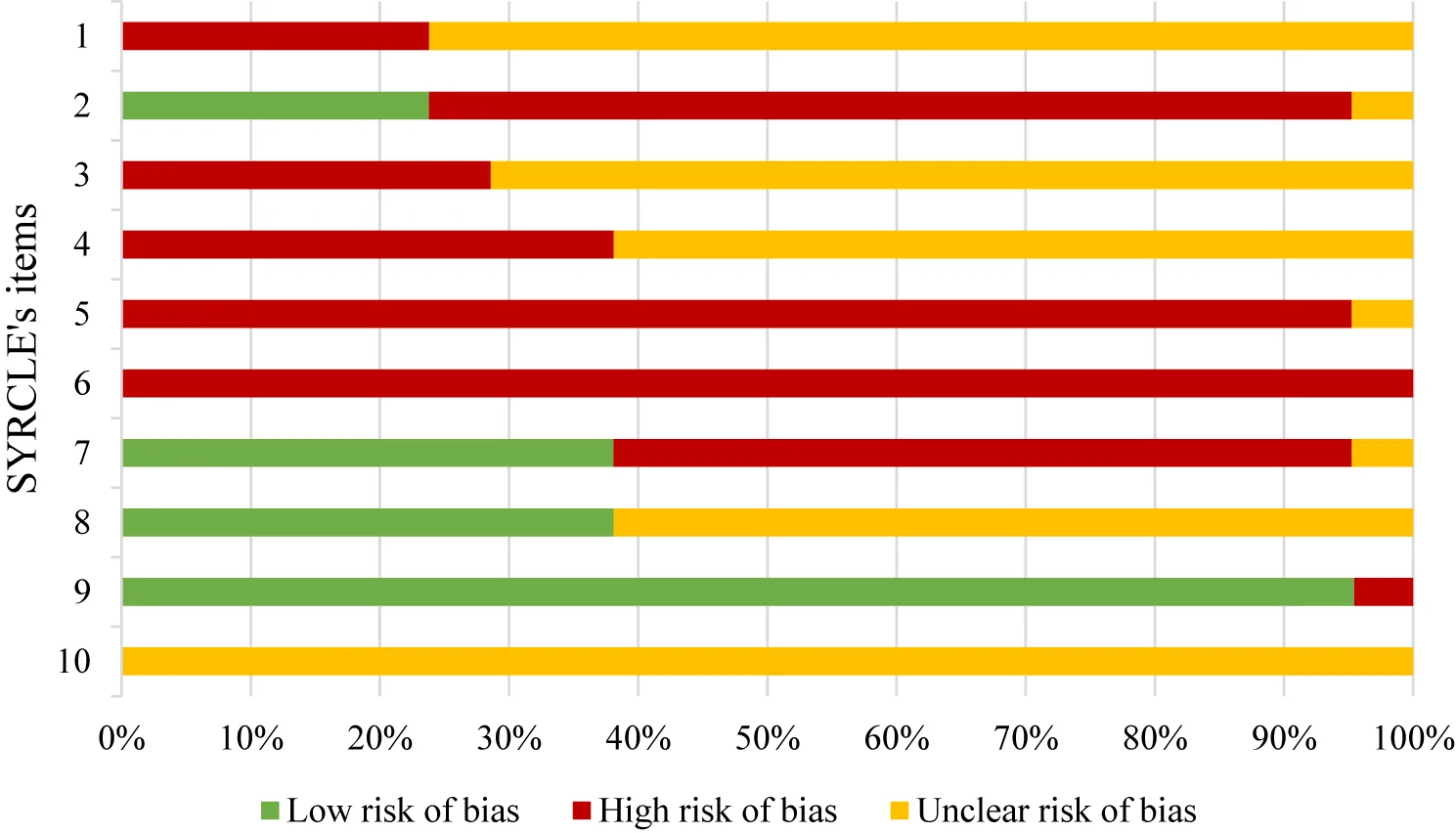
Frequencies (%) of risk of bias assessment according to systematic review centre for laboratory

The appraisal performed using the SYRCLE’s RoB tool revealed greater frequencies of high and uncertain risk, as illustrated in Fig. 2. The included studies met at least three items of SYRCLE’s RoB tool. Most of the included studies did not mentioned methods for blinding the investigators during the experiment or randomizing the selection of the animals to evaluate the outcomes. Moreover, a large part of the studies failed to report clearly how the allocation sequence of the animal models was generated, applied and concealed, how relevant characteristics were standardized for the treatment and control groups, how the randomization was performed in the lodging of the animals during the experiment or whether the study was apparently free of other problems that could result in biases. The summary of the methodological quality assessment of all studies is presented in Table 3.

**Table 3:** SYRCLE’s Rob toll criteria for quality assessment

Author	Year	Selection			Performance		Detection		Attrition	Reporting	Other	Yes items
		1	2	3	4	5	6	7	8	9	10	
Ahmad et al.	2020	Unclear	No	Unclear	Unclear	No	No	No	Unclear	Yes	Unclear	1/10
Almasry et al.	2014	Unclear	No	Unclear	Unclear	No	No	Yes	Yes	Yes	Unclear	3/10
Arican et al.	2018	Unclear	No	Unclear	No	Unclear	No	Unclear	Unclear	Yes	Unclear	1/10
Asjid et al.	2018	No	No	No	Unclear	No	No	No	Unclear	Yes	Unclear	1/10
Chouhan et al.	2019	Unclear	Yes	No	No	No	No	No	Unclear	Yes	Unclear	2/10
Coskun et al.	2018	Unclear	Yes	Unclear	Unclear	No	No	No	Yes	Yes	Unclear	3/10
Gamal et al.	2019	No	No	No	No	No	No	No	Unclear	Yes	Unclear	1/10
Guner and Buyukbebeci	2013	Unclear	No	Unclear	Unclear	No	No	No	Yes	Yes	Unclear	2/10
Hermeto et al.	2016	Unclear	No	Unclear	Unclear	No	No	Yes	Unclear	Yes	Unclear	2/10
Jayaram et al.	2020	Unclear	No	Unclear	Unclear	No	No	Yes	Unclear	Yes	Unclear	2/10
Ji et al.	2015	Unclear	No	Unclear	Unclear	No	No	No	Yes	Yes	Unclear	2/10
Kanwat et al.	2017	No	Yes	No	No	No	No	Yes	Unclear	Yes	Unclear	3/10
Kutuk et al.	2014	No	No	No	Unclear	No	No	Yes	Yes	Yes	Unclear	3/10
Lu et al.	2020	Unclear	Yes	Unclear	Unclear	No	No	No	Yes	Yes	Unclear	3/10
Parlak and Arican	2020	Unclear	No	Unclear	No	No	No	No	Unclear	Yes	Unclear	1/10
Ragab et al.	2021	Unclear	Yes	Unclear	Unclear	No	No	Yes	Unclear	Yes	Unclear	3/10
Wang et al.	2018	Unclear	Unclear	Unclear	No	No	No	Yes	Yes	Yes	Unclear	3/10
Wu et al.	2016	Unclear	No	Unclear	Unclear	No	No	No	Yes	Yes	Unclear	2/10
Xin et al.	2020	Unclear	No	Unclear	No	No	No	No	Unclear	Yes	Unclear	1/10
Yin et al.	2016	Unclear	No	Unclear	Unclear	No	No	No	Unclear	Yes	Unclear	1/10
Yun et al.	2016	No	No	No	No	No	No	Yes	Unclear	Yes	Unclear	2/10

*Note* YES answers indicated low risk of bias, NO indicated high risk of bias, and UNCLEAR indicated it was not possible to assign bias. Criteria used for publication risk of bias analysis: (1) Was the allocation sequence adequately generated and applied? (2) Were the groups similar at baseline or were they adjusted for confounders in the analysis? (3) Was the allocation adequately concealed? (4) Were the animals randomly housed during the experiment? (5) Were the caregivers and/or investigators blinded from knowledge which intervention each animal received during the experiment? (6) Were animals selected at random for outcome assessment? (7) Was the outcome assessor blinded? (8) Were incomplete outcome data adequately addressed? (9) Are reports of the study free of selective outcome reporting? (10) Was the study apparently free of other problems that could result in high risk of bias?

### Comparison of treatment with PRP versus control

#### Cartilage histology

**Fig. 3:**
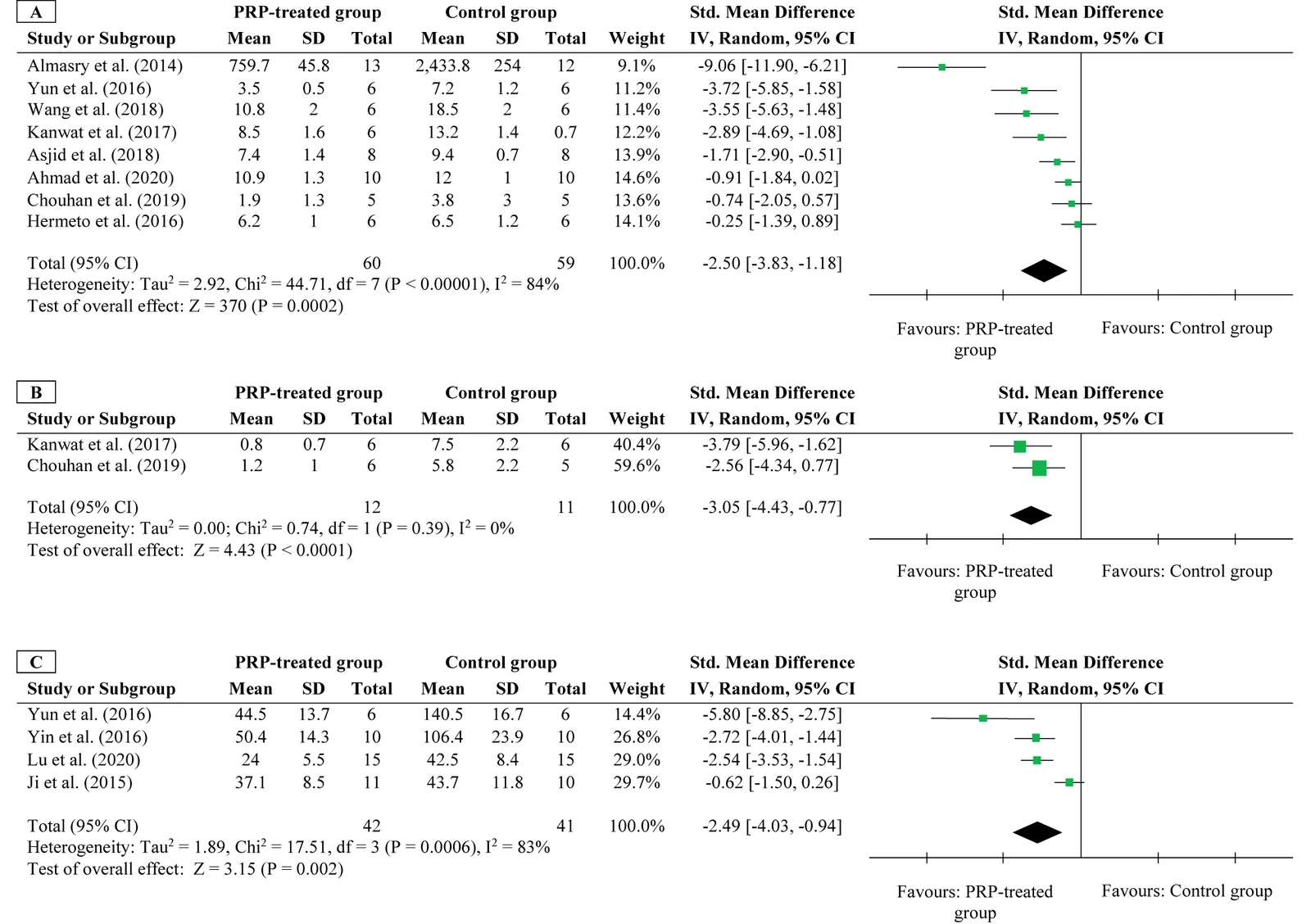
Forest plots for effect of the intra-articular injection of PRP on (**A**) cartilage, (**B**) synovium, (**C**) IL-1 levels, (**D**) IL-6 levels, (**E**) TNF-α levels and (**F**) PDGF-A levels

The meta-analysis of eight studies [16, 25, 37, 41, 45, 46, 48, 52] indicated that treatment with PRP achieved superior results compared to the control for changes in cartilage, as the samples from these studies presented less cartilage damage after treatment (pooled sample of 119 animals [goat, dog, guinea pig, rabbit and rat]; very low quality of evidence [items met: inconsistency and publication bias]; SMD = −2.50 [large effect]; 95% CI: −3.83 to −1.18; *p* = 0.0002; *I*^*2*^ = 84% [high heterogeneity] and control groups received either placebo or no type of treatment) (Fig. 3A).

#### Synovium histology

The meta-analysis of two studies [37, 48] indicated that treatment with PRP achieved superior results compared to the control for changes in synovium, as the samples from these studies presented less synovitis (combined sample of 23 animals [guinea pigs]; very low quality of evidence [items met: inconsistency and indirectness]; SMD = −3.05 [large effect]; 95% CI = −4.43 to −0.77; *p* < 0.0001; *I*^*2*^ = 0% [small heterogeneity] and control groups received no type of treatment) (Fig. 3B).

#### Interleukin (IL)-1 levels

The meta-analysis of four studies [36, 39, 44, 45] indicated that treatment with PRP achieved superior results compared to the control regarding the concentration of IL-1, as the samples from these studies presented a lower concentration of this proinflammatory interleukin (combined sample of 83 animals [dog and rabbit]; low quality of evidence [items met: inconsistency, indirectness and publication bias]; SMD = −2.49 [large effect]; 95% CI = −4.03 to −0.94; *p* = 0.002; *I*^*2*^ = 83% [high heterogeneity] and control groups received no type of treatment) (Fig. 3C).

#### Interleukin-6 levels

The meta-analysis of three studies [25, 36, 39] indicated that treatment with PRP achieved superior results compared to the control regarding the concentration of IL-6, as the samples from these studies presented a lower concentration of this proinflammatory interleukin (combined sample of 71 animals [rabbit and rat]; very low quality of evidence [items met: inconsistency and publication bias]; SMD = −3.76 [large effect]; 95% CI = −5.37 to −2.14; *p* < 0.00001; *I*^*2*^ = 73% [moderate heterogeneity] and control groups received no type of treatment) (Fig. 3D).

#### Tumor necrosis factor alpha (TNF-α) levels

The meta-analysis of four studies [25, 36, 39, 45] indicated that treatment with PRP achieved superior results compared to the control for the concentration of TNF-α, as the samples from these studies presented a lower concentration of this pro-inflammatory cytokine (combined sample of 83 animals [dog, rabbit and rat]; very low quality of evidence [items met: inconsistency and publication bias]; SMD = −3.70 [large effect]; 95% CI = −5.19 to −2.22; *p* < 0.00001; *I*^*2*^ = 72% [moderate heterogeneity] and control groups received no type of treatment) (Fig. 3E).

#### Platelet-derived growth factor (PDGF)

The meta-analysis of two [46, 50] studies indicated that treatment with PRP did not obtain superior results compared to the control for the concentration of PDGF-A (combined sample of 35 animals [rat]; very low quality of evidence [items met: inconsistency and publication bias]; SMD = −1.51 [large effect]; 95% CI = −14.09 to 11.06; *p* = 0.81; *I*^*2*^ = 96% [high heterogeneity] and control groups received no type of treatment) (Fig. 3F).

## Discussion

The aim of this systematic review with meta-analysis was to investigate the effect of the intra-articular injection of PRP on inflammatory process and histopathological characteristics of cartilage and synovium in animal models with OA. As the main and innovative result, the PRP treatment led to less cartilage damage and synovitis as well as reductions in the concentration of the proinflammatory markers IL-1, IL-6 and TNF-α in animal models. Based on the GRADE approach, the quality of the evidence assessed was low to very low. The effect size in the meta-analyses was large.

The level of evidence for both cartilage and synovium histology was very low with a large effect (*p* = 0.0002 [16, 25, 37, 41, 45, 46, 48, 52], and *p* < 0.0001 [37, 48], respectively). The Modified Mankin Score for cartilage and the Pelletier Score for synovium were employed, reporting lower means in comparison to the CG, indicating reduced cartilage damage and synovitis post-PRP treatment. For cartilage histology, PRP dosage ranged from 0.5 mL [16, 42] to 1 mL [45], and administration protocols included single [16] and weekly applications for three, four, and six weeks [37, 42, 45, 48]. Notably, shorter treatment protocols, such as a single PRP application, proved effective in minimizing cartilage damage caused by OA. For synovium histology, however, neither Chouhan et al. [48] nor Kanwat et al. [37] specified the dosage of PRP administrated, and both employed a single weekly application for three weeks. Although the meta-analysis exhibited low heterogeneity (0%), noteworthy is that both studies are from the same research group, contributing to the lowered evidence level (publication bias per the GRADE approach).

The evidence levels for IL-1, IL-6, and TNF-α concentrations after PRP treatment varied, with low evidence for IL-1 (*p* = 0.002) [36, 39, 44, 45] and very low evidence for IL-6 and TNF-α (*p* < 0.00001 [25, 36, 39], *p* < 0.00001 [25, 36, 39, 45], respectively). ELISA was the primary assessment method, consistently showing lower means post-PRP treatment, indicating reduced inflammatory processes. PRP dosage and treatment protocols varied across studies. For IL-1, PRP dosage ranged from 0.5 mL [36, 39] to 3 mL [44], and treatment protocols varied from weekly applications spanning three [44] to ten weeks [39]. In the case of IL-6, PRP dosage ranged from 0.5 mL [36, 39] to 1 mL [25], and treatment protocols ranged from a single injection [25] to five [36] or ten [39] weekly applications. Similarly, for TNF-α, PRP dosage ranged from 0.5 mL [36, 39] to 1 mL [25, 45], and treatment protocols ranged from a single injection [25] to four [45], five [36], or ten [39] weekly applications. Shorter treatment protocols, whether spanning three weeks for IL-1, or a single injection for IL-6 and TNF-α, demonstrated effectiveness in reducing concentrations across these inflammatory markers.

Concerning PDGF-A levels however, the findings indicate very low-quality evidence with a large effect (*p* = 0.81) [46, 50] suggesting that PRP is not superior to sham. Gamal et al. [50] observed a lower mean in the PRP-treated groups, whereas Almasry et al. [46] reported a higher mean, indicating elevated PDGF-A levels following PRP treatment. The administered PRP doses were 0.2 and 0.085 mL, respectively, with both studies employing a consistent application period of once per week for three weeks.

While this review enhances our understanding of the histological effects on cartilage and synovium following PRP injection, it is crucial to interpret these findings with caution. The majority of the evidence assessed through the GRADE approach was rated as very low, emphasizing the need for careful consideration and acknowledgment of potential limitations. It is also important to highlight that the experimental heterogeneity among the included studies was one of the main reasons for lowering the quality of the evidence. Furthermore, high heterogeneity was found for the histology of cartilage and synovium, as well as inflammatory markers such as IL-1, IL-6, and TNF-α. This high heterogeneity may be due to the different species of animals, the different PRP preparation methods, and the intervention protocols used by the studies (experimental heterogeneity). Even though most meta-analyses show high heterogeneity, our results are in agreement with the literature, given that about 25% of the meta-analyses developed present *I*^*2*^ values above 50% [34].

Meta-analyses of randomized clinical trials (RCTs) have suggested that PRP treatments improve pain and function in knee and hip OA patients [56, 57]. However, recent RCTs [58, 59] contradict these findings, showing no benefit of PRP over placebo. The meta-analyses, conducted prior to these RCTs, may have been compromised not only by comparisons with no first line treatments for knee OA like hyaluronic acid and corticosteroids but also by the inclusion of studies with low levels of evidence and methodological rigor. This could have significantly affected the evaluation of PRP efficacy. While our study observed reductions in proinflammatory markers following PRP treatment, the clinical significance, particularly regarding long-term joint health, remains uncertain given the conflicting evidence on PRP’s efficacy for pain and function improvement.

The appraisal of the methodological quality of the studies was performed using the SYRCLE’s’ RoB tool to determine the risk of bias and the GRADE approach was used to determine the quality of the evidence. Among the ten items on the SYRCLE scale, the criteria with the greatest risk of bias were item 5 (blinding of the investigators) and item 6 (randomization of animal selection for the evaluation of the outcomes). None of the studies included fulfilled the item 6. Regarding item 1, sixteen studies reported randomization during the generation of the allocation sequence, but none described the method used. Although the majority of studies reported apparent similarities in the baseline characteristics of the animal models, such as weight, age, and sex, only five studies [26, 37, 39, 48, 49] provided details on tests performed to determine statistical differences in these characteristics within the sample.

Regarding the GRADE analysis, the indirectness and imprecision were the main items responsible for lowering the quality of the evidence. There was a variety of tests used to assess outcomes, and different species of animals were used. This reason negatively affected item 3 (indirectness) of the GRADE approach. The sample size was low in all studies included. To fulfill item 4 (imprecision) from the GRADE approach, the sample size from all the included studies must reach over 200 animals by comparison groups. Therefore, more studies are needed.

The Modified Mankin Score was used in seven studies [16, 25, 37, 41, 45, 48, 52] and only one of these did not find a significant difference after the treatment [52], with a small effect size encountered. The six studies [16, 25, 37, 41, 45, 48] found significant differences and a large effect size. Thus, if Hermeto et al. [52] had increased the sample size, it is possible that the authors would have found a significant difference with large effect size.

This is the first study to synthesize evidence regarding the effects of PRP on the inflammatory process as well as histological characteristics of the cartilage and synovium in animals with OA. The type of treatment selected (PRP) proved to be promising for animals with OA and needs to be investigated better. Another strong point was the methodology adopted, which followed the PRISMA guidelines and recommendations of the Cochrane Collaboration and involved the GRADE approach. These points allowed a qualitative and quantitative analysis of the results. However, we must recognize divergence with regards to the sample (we had to pool the animals for the assessment of the quality of evidence through GRADE approach), the OA induction method, the different PRP preparation protocols and doses, the period and frequency of the applications and the measurement tools employed for the evaluation of the outcomes. We hope that our study would be of some help in the design of high-quality preclinical studies in the future.

## Conclusions

Treatment with PRP seems to be capable of lowering concentrations of proinflammatory markers such as IL-1, IL-6 and TNF-α (very low level of evidence with a large effect) and cartilage and synovium damage (low level of evidence with a large effect) in animals with OA. Further studies with greater methodological rigor and larger samples are needed to improve the quality of evidence.

## Data Availability

The datasets used and/or analysed during the current study available from the corresponding author on reasonable request.

## Abbreviations

CI: Confidence interval
GAG: Glycosaminoglycan
GFs: Growth factors
GRADE: Grading of recommendations, assessment, development and evaluations
IL: Interleukin
MeSH: Medical subject headings
OA: Osteoarthritis
PDGF: Platelet-derived growth factor
PRISMA: Preferred reporting items for systematic reviews and meta-analyses
PROSPERO: International prospective register of systematic reviews
PRP: Platelet-rich plasma
RoB: Risk of bias
SMD: Standardized mean differences
StArt: State of the art through systematic review
TNF-α: Tumor necrosis factor alpha
